# Reformulating the susceptible–infectious–removed model in terms of the number of detected cases: well-posedness of the observational model

**DOI:** 10.1098/rsta.2021.0306

**Published:** 2022-10-03

**Authors:** Eduard Campillo-Funollet, Hayley Wragg, James Van Yperen, Duc-Lam Duong, Anotida Madzvamuse

**Affiliations:** ^1^ Department of Statistical Methodology and Applications, School of Mathematics, Statistics and Actuarial Science, University of Kent, Canterbury, Kent CT2 7PE, UK; ^2^ Department of Engineering Mathematics, School of Computer Science, Electrical and Electronic Engineering and Engineering Maths, University of Bristol, Bristol BS8 1TW, UK; ^3^ Department of Mathematics, School of Mathematical and Physical Sciences, University of Sussex, Brighton, East Sussex BN1 9QH, UK; ^4^ School of Engineering Science, LUT University, Lappeenranta 53850, Finland; ^5^ Department of Mathematics, University of Johannesburg, Johannesburg, South Africa; ^6^ University of British Columbia, Department of Mathematics, Vancouver, Canada

**Keywords:** susceptible–infectious–recovered, epidemiology, existence, uniqueness, observational model

## Abstract

Compartmental models are popular in the mathematics of epidemiology for their simplicity and wide range of applications. Although they are typically solved as initial value problems for a system of ordinary differential equations, the observed data are typically akin to a boundary value-type problem: we observe some of the dependent variables at given times, but we do not know the initial conditions. In this paper, we reformulate the classical susceptible–infectious–recovered system in terms of the number of detected positive infected cases at different times to yield what we term the observational model. We then prove the existence and uniqueness of a solution to the boundary value problem associated with the observational model and present a numerical algorithm to approximate the solution.

This article is part of the theme issue ‘Technical challenges of modelling real-life epidemics and examples of overcoming these’.

## 1. Introduction

At the beginning of any pandemic, mathematical modelling of infectious diseases is thrust into the limelight for governments across the globe to gain imperative forecasting pictures of the impending spread of the infectious disease, as demonstrated by the 2009–2010 swine flu pandemic [1–4], the 2015–2016 Zika virus epidemic [5–8] and the most recent COVID-19 pandemic [9–12]. Non-pharmaceutical interventions (NPIs) are interpreted by adjusting model parameters to allow for quick decision-making on the appropriate NPI measure(s) to be implemented. Understanding and predicting levels of infections in the community is the crux of mathematical modelling in epidemiology since it is these infections that drive the epidemic and also the mathematical models. However, getting accurate, reliable data to describe the current number of infections within the community is arguably the largest challenge for data-driven modelling. Not only is lack of data problematic when deriving and using mathematical models, the context and interpretation of the data and the parameter fitting process often comes with difficulties. In particular, there is often not a one-to-one correspondence between data and parameters or compartments in a model, especially when considering daily snapshots of an epidemic, for example.

In this work, we study the susceptible–infectious–removed (SIR) equations often accredited to the work of Kermack & McKendrick in 1927 [13]. In particular, we look to demonstrate how to use the number of detected cases directly in the model formulation and prove the existence and uniqueness of a solution to the resulting formulation. The SIR equations take the following form of a simple temporal dynamic system of ordinary differential equations, supported by non-negative initial conditions

1.1 $$\begin{array}{rl} \dot{S} & =-\beta \frac{I}{N}S,\;S(0)=S_{0}, \end{array}$$

1.2 $$\begin{array}{rl} \dot{I} & =\beta \frac{I}{N}S-\gamma I,\;I(0)=I_{0}, \end{array}$$

**Figure 1.**
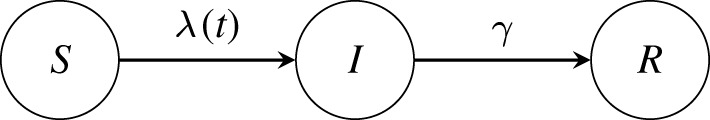
Schematic of the SIR compartmental model. We let S denote the proportion of the total population N who are susceptible to the infectious disease being studied. Susceptible individuals become infectious with the disease to form the I subpopulation at a rate λ(t), which represents the current average infection rate. The rate λ(t) is the product of β, the average transmission rate, and the probability of meeting an infectious person $I(t)\;N^{-1}$. Individuals in the I(t) subpopulation then are removed at a rate γ to form the removed subpopulation R. The description of the removed compartment depends on the nature of the infectious disease and the interpretation of the data available to be used. Typically, the term removed is interchanged with the term recovered. As is standard for epidemiological models of this nature, $\beta^{-1}$ denotes the average time between transmissions and $\gamma^{-1}$ denotes the average length of time before removal.

1.3 $$\begin{array}{rl} \;\mathrm{and}\;\dot{R} & =\gamma I,\;R(0)=R_{0}, \end{array}$$

where the dot above the notation denotes the time derivative, see figure 1 for a schematic. As is standard for the SIR equations, we let N denote the total population being considered, β denote the average transmission rate and γ denote the average removal rate. The initial conditions are chosen so that $S_{0}+I_{0}+R_{0}=N$, which gives rise to S(t)+I(t)+R(t)=N for any t≥0. Moreover, it is standard to make the assumption that $I_{0}\geq 1$, i.e. there is at least one infected individual at the onset of the infectious disease. We direct readers to the following literature to gain insight into the analysis and uses of the SIR equations and its family of compartmental models [14–17].

The ‘observational model’, as coined in [18], is a representation of a compartmental model, such as the SIR equations, described in terms of the parameters and compartments that are captured, or to be inferred, by the mathematical interpretation of the data. This observational model gives an intuitive understanding of what parameters can be identified and how the data affects different compartments in the model. Typically, data for infectious diseases come as a daily, or in general, periodic, snapshot of some of the states that individuals experience during the infectious disease. In this study, we are looking to use the detected cases of an infectious disease, which we denote by $X_{m}$. In practical terms, we are assuming that our observation is a fraction r∈(0,1) of the total number of individuals leaving the infectious state I over the time of the observation. This situation arises when the individual ceases to be infectious once they have been diagnosed, for example because the individual can avoid contact with susceptible individuals. We allow for the situation that we only observe a fraction r of cases since there are situations where not all infectious individuals can be removed, such as false-negatives in testing regimes, bad individual compliance in behaviour or financial/resource restrictions on the amount of testing that can be done. In reality, r is likely to be time-dependent since it can characterize aspects of public policy, however, we keep it constant in this study. In order to describe these observations in terms of the model parameters and compartments, we formally describe the removed compartment R as those who are no longer mixing in the general population. Hence, for each observation of the number of detected cases $X_{m}$, m=0,…,M, we see that

1.4 $$X_{m}:=r\gamma \int_{t_{m}}^{t_{m+1}}I(s)\;\text{d}s,$$

where $\left[t_{m},t_{m+1}\right]$ represents the time interval of the measurement of the data. This definition of the observed data for the number of detected cases of an infectious disease has been used in the past; see for instance [19].

Using (1.4) as the interpretation of the available data leads to the observational model

1.5 $$\ddot{I}=\dot{I}\left(\frac{\dot{I}}{I}-\beta \frac{I}{N}\right)-\beta \;\gamma \frac{I^{2}}{N},$$

a second-order nonlinear differential equation equipped with non-local boundary conditions (1.4), see §3 for the detailed derivation. A significant part of this work is devoted to studying the boundary value problem (1.4) and (1.5). In contrast to initial value problems that typically arise when studying mathematical epidemiology, boundary value problems are much more difficult to deal with. For instance, it is well known that a small change in the boundary conditions may lead to significant changes in the behaviour of the solutions [20]. In fact, there is no general theory to the existence and uniqueness of a solution for boundary value problems. In most instances, we have to study the boundary value problem on a case-by-case basis, especially when the problem at hand is nonlinear, and/or the boundary conditions are atypical, which is the case with our problem as the boundary condition (1.4) is given in a non-local integral form. Non-local boundary conditions have been studied in the past in various forms, recently appearing in the study and application of fractional differential equations [21]. In our case, the non-local boundary conditions correspond to fixing the values of certain integrals of the solution over given time intervals, which is similar to how the average value is imposed on certain boundary value problems with Neumann boundary conditions to ensure uniqueness of the solution. Although this form is convenient for the analysis, we note that we can convert the integral conditions to nonlinear, multi-point Robin-type conditions. This, however, is only possible due to the set-up of the observational model and thus the problem at hand, since the differential equation directly involves the data, and thus the boundary condition in some capacity. As far as we are aware, this is the first work to consider a boundary value problem with these specific integral-type boundary conditions. The key message of our study is on the transformation of an epidemiological initial value problem to a boundary value problem due to the appropriate form of the observed data, hence we refer readers to [20–26], and the references therein, for further information on methods and techniques that may be used to analyse certain classes of boundary value problems with different types of boundary conditions.

The main results of this publication can be summarized in the following two theorems:

**Theorem 1.1 (Existence).**

*Let* $\beta_{e}:=r^{-1}\beta \;N^{-1}$. *Given* $X_{0},X_{1}>0$ *corresponding to (1.4), and given parameters* $\beta_{e}$, γ>0, *there exists a solution* $I\in C^{2}(t_{0},t_{2})$ *to (1.5) subject to (1.4)*.

**Theorem 1.2 (Uniqueness).**

*Let* $\beta_{e}:=r^{-1}\beta \;N^{-1}$. *Given* $X_{0},X_{1}>0$ *corresponding to (1.4), and given parameters* $\beta_{e}$, γ>0, the *solution to (1.5) subject to (1.4) is unique in* $C^{2}(t_{0},t_{2})$. *Furthermore, the solution is smooth*.

The structure of the paper is as follows: in §2, we motivate how the approach outlined in this work came about and why it is necessary, in §3, we derive the observational model and pose an equivalent boundary value problem to (1.5), in §4, we prove Theorem 1.1, in §5, we prove theorem 1.2 and in §6, we outline an algorithm for the numerical solution to the observational model with two examples.

## 2. Motivation

Since the onset of COVID-19, the disease caused by SARS-CoV-2, in December 2019 and the declaration of a pandemic by the World Health Organization in March 2020, governments and organizations across the globe have implemented unprecedented regional, national and international interventions to contain the spread of COVID-19 and the subsequent damage caused by it. Initially, NPIs were the only tool in the fight to contain the spread of the disease, forcing governments to introduce strict control measures on public mixing, the use of personal protective equipment, improvement of personal hygiene and the use of contact tracing to reduce the possibility of transmission throughout their countries, protecting those most vulnerable as well as maintaining control of the healthcare demand and capacity. The social-economical effects of these necessary decisions are still unknown, and it is speculated that these effects will still be felt for many more years to come [27–30]. The global picture is starting to look less bleak with the introduction of effective vaccines and vaccination policies allowing the relaxation of such strict NPIs, however with the spawn of new variants of COVID-19, such as the Alpha variant (lineage B.1.1.7) and more recently the Delta variant (lineage B.1.617.2), it is clear we are not out of the pandemic yet [31–34]. Indeed, now that ‘normality’ has returned, country borders have opened and tourism is on the increase, we in the UK are amidst an increase in daily positive cases, hospitalizations and deaths, while other European countries are tightening their COVID-19 restrictions or even going back into lockdowns.

In response to the pandemic in the Sussex region of the UK, local authority organizations in Sussex created a consortium that posed modelling questions of strategic operational significance to the Local Health Resilience Partnership covering East Sussex, West Sussex and Brighton & Hove. As a result, leaders from Public Health Intelligence in the local authority organizations approached the University of Sussex to undertake COVID-19 epidemiological modelling that is specific to Sussex as the national level modelling from the Scientific Advisory Group for Emergencies was not applicable at the regional, integrated care level. In response to their request, we developed a data-driven SIR-type mathematical model, which in practice has been used to answer public health questions based around healthcare demand and capacity, and mortuary capacity, such as the impact of second waves, future lockdowns and vaccination supply [18]. In our early parameter estimation attempts, it became clear to us that the data provided were not compatible with the standard SIR-type formulation, in particular, the data did not provide an accurate estimation of all the initial conditions of the compartments of the model. Healthcare data typically come in one of three types: new daily data (such as admissions), current daily data (such as beds occupied) or cumulative daily data (such as total number of deaths). Due to the methods and constraints on data collection, data typically come as new daily data, i.e. a change in the state of an individual. For example, being admitted into hospital is a change of state from being infectious in the community to being in a hospital bed, whereby the infectious state and hospital bed state are compartments of a mathematical model, while the admitted component is the change between compartments. In this set-up, the admissions data are proportional to the infectious compartment, but not directly as it is an integral, so identifying the initial conditions of the compartments from the data becomes a real challenge, see [18] for more details.

Although the remit of this paper does not include parameter estimation results, we nevertheless describe these in order to demonstrate the issue of the estimation of initial conditions described above. Given that we have a system of nonlinear differential equations, the parameter estimation problem can be stated as a nonlinear least-squares (NLS) optimization problem [35]. Let d describe the observed data, θ:=[β,γ] describe the set of parameters we are estimating, x(θ):=[S(θ),I(θ)] describe the set of solutions to (1.1) and (1.2) with f(x(θ),θ) describing the differential equations, and y(x,θ) denote the function that maps the solutions x to the mathematical interpretation of the data. Let L(d|θ;y(x,θ)) describe the likelihood function associated with the probability distribution assumption of the noise between d and y. Then, the NLS optimization problem can be stated as

$$\begin{array}{rl} {\text{maximize}}_{\theta }\; & L(d|\theta ;y(x,\theta ))\\ \text{subject to}\; & \theta \in D,\\ & \dot{x}(\theta )=f(x(\theta ),\theta ),\\ & x(0)=x_{0}, \end{array}$$

where D denotes the admissible parameter space. The NLS problem easily demonstrates the issue of the initial conditions faced: that even although parameters θ can be estimated regardless of the initial conditions, they may not be representative of real life. This impacts the use of epidemiological models, that they may not have any accurate realistic forecasting capabilities. Moreover, it can easily be reasoned that L is highly non-convex with respect to the parameters and in practice will therefore likely converge to solutions close to the initial guess, which may not be the global solution [36,37].

The standard approach in the literature to navigate the issue of estimating initial conditions is to restrict the parameters, for example, by fixing the basic reproductive number, and estimate when the epidemic began using the data provided, see for instance [38]. Fortunately for modellers in the UK, with regards to COVID-19, the disease was brought to us from outside our borders and so identifying ‘patient zero’ and tracing their connections becomes easier. However, with epidemiological data in particular, what is often not considered is that there is stochastic perturbation in the realization of the actual epidemic. The spread of an infectious disease is a stochastic process, and so the collection of data from an epidemic is just one realization of that process. Noise in the data presents itself mathematically as uncertainty in the parameters, while stochastic perturbation in the epidemic presents itself mathematically as uncertainty in both the initial conditions and the parameters. Variability due to stochastic perturbation is high at the onset of an epidemic, especially in a spatially complex and heterogeneous population not typically accounted for in modelling, which makes estimating the first infection and calibrating to data difficult. In [18], we took an alternative approach and estimated the initial conditions from the data at the onset of the first national lockdown, since this was the first available bit of data. In this instance, the initial conditions became part of the parameters we looked to estimate, however this proved challenging due to the nonlinearity of the NLS problem and the differences in parameter sensitivity between initial conditions and system parameters (such as β and γ). This led us onto the problem we are trying to solve currently: Can we incorporate the data into the model formulation in a more natural way that overcomes the issue of accurate estimation of initial conditions? We believe that by rewriting the SIR equations as the observational model with boundary conditions, we can gain accurate estimates of parameters while bypassing issues with initial conditions. However, before approaching the parameter identifiability and estimation problems, we first need to demonstrate the non-trivial well-posedness of the observational model.

## 3. Derivation of the observational model

Before we derive the observational model, it is important we provide some standard analytical results of (1.1) and (1.2). It is a standard result in dynamical systems and phase space analysis that for positive initial conditions, we have 0<S(t)<N and 0<I(t)<N for t≥0. In particular, steady-state analysis tells us that

$$\lim_{t\to \infty }(S(t),I(t),R(t))=(S_{\infty },0,N-S_{\infty }),$$

where $S_{\infty }>0$. Due to (1.1), S(t) is monotonically decreasing, which implies that

3.1 $$\lim_{I\to 0}\frac{\dot{I}(t)}{I(t)}=\lim_{I\to 0}\beta \frac{S(t)}{N}-\gamma =\beta \frac{S_{\infty }}{N}-\gamma .$$

In addition, since the right-hand sides of (1.1) and (1.2) are smooth functions, their solutions are infinitely differentiable (see, for instance, [39]), which allows us to make the necessary steps to derive the observational model. Turning our attention to the data (1.4), for the analysis, we assume that, for any m=0,…,M,

$$0<X_{m}<\infty .$$

That is to say that we always observe a strictly positive, finite number of cases since we are not considering any observation error or model error in this study, i.e. our observations are exact. Indeed, in this situation, the case $X_{m}=0$ implies that there no more infectious cases in the population, and in consequence, there will be no more cases in future observations.

As described in [18], since the data (1.4) are described by the infectious compartment I, we need to rewrite equations (1.1) and (1.2) to be purely in terms of I in order to derive the observational model. To this end, we note three properties of equations (1.1) and (1.2), namely

3.2 $$\dot{S}+\dot{I}=-\gamma I,$$

which, by differentiating, immediately gives us

3.3 $$\ddot{S}+\ddot{I}=-\gamma \dot{I}.$$

Moreover, by differentiating (1.1), we see that

3.4 $$\ddot{S}=\dot{S}\left(\frac{\dot{I}}{I}-\beta \frac{I}{N}\right)$$

and hence, inserting (3.2) and (3.3) into (3.4), the observational model takes the form

$$\ddot{I}=\dot{I}\left(\frac{\dot{I}}{I}-\beta \frac{I}{N}\right)-\beta \;\gamma \frac{I^{2}}{N},$$

as seen in (1.5). One notes that (3.4) and thus (1.5) are well defined for t≥0 due to (3.1). For the purposes of this paper, we continue to transform (1.5) to facilitate the analysis. By denoting $\beta_{e}:=r^{-1}\beta \;N^{-1}$ and setting z(t)=ln⁡(r γ I(t)), (1.5) is equivalent to

3.5 $$\ddot{z}=-\beta_{e}\;e^{z}\left(\frac{\dot{z}}{\gamma }+1\right),$$

while the data (1.4) are equivalent to

3.6 $$X_{m}=\int_{t_{m}}^{t_{m+1}}\;e^{z(s)}\;\text{d}s.$$

In the subsequent sections, we will prove the following two theorems, which are respectively equivalent to Theorems 1.1 and 1.2 due to the continuous bijective transformation between z and I.

**Theorem 3.1 (Existence).**

*Given* $X_{0},X_{1}>0$, *corresponding to (3.6), and given parameters* $\beta_{e},\gamma >0$, *there exists a solution* $z\in C^{2}(t_{0},t_{2})$ *to (3.5) subject to (3.6)*.

**Theorem 3.2 (Uniqueness).**

*Given* $X_{0},X_{1}>0$ *corresponding to (3.6), and given parameters* $\beta_{e},\gamma >0$, *the solution to (3.5) subject to (3.6) is unique in* $C^{2}(t_{0},t_{2})$. *Furthermore, the solution is smooth*.

**Remark.**

One can observe that as I tends to 0, z becomes negatively unbounded. This is not impactful on the analysis due to the boundary condition (3.6), equivalently (1.4), always being positive. As we noted above, in the situation that (3.6) is zero, and upon assuming no noise in the data, this implies that the epidemic is over and thus this model is not relevant anymore.

**Remark.**

One can go further to simplify (3.5) by taking $w(\xi )=z(t)+\ln(\beta_{e}\gamma^{-2})$, where ξ=γt, to get

$$w^{\prime \prime }=-e^{w}(w^{\prime }+1),$$

where the prime denotes the derivative with respect to ξ, which leads to (3.6) satisfying

$$X_{m}=\frac{\gamma }{\beta_{e}}\int_{\gamma t_{m}}^{\gamma t_{m+1}}\;e^{w(\eta )}\;\text{d}\eta .$$

**Remark.**

To continue to demonstrate the issues with initial conditions, we can express S(0) and I(0) in terms of z and the parameters. By definition of z, we obtain

3.7 $$S(0)=N\frac{\dot{z}(0)+\gamma }{\beta }$$

and

3.8 $$I(0)=\frac{e^{z(0)}}{r\gamma }.$$

From the formulation of the observational model (3.5), we can see that a solution z is defined only in terms of $\beta_{e}$ and γ. Assuming that N is known, due to the definition of $\beta_{e}$, the pair (β,r) cannot be uniquely identified from the data; in other words, for a given $\beta_{e}$, any pair (β,r) satisfying $\beta_{e}=r^{-1}\beta \;N^{-1}$ will produce exactly the same solution. This implies that in order to simulate future predictions with the original SIR formulation, one would need estimates for r, as well as the initial conditions. Indeed, from (3.7), we conclude that it is not possible to identify S(0) unless both β and r are known; in particular, since it is not possible to identify both β and r from the data, it is not possible to recover the initial conditions without an independent estimate of the under-reporting coefficient r. By contrast, an advantage of the formulation of the observational model is that it will allow us to compute the solution z, and predict future observations $X_{m}$, as long as we can fit the model to identify the value of $\beta_{e}$. Note also that, without an independent estimate on r, we can only infer the ratio between the initial number of infectious individuals to the initial number of susceptible individuals since

$$\frac{I(0)}{S(0)}=\frac{\beta_{e}}{\gamma }\frac{e^{z(0)}}{\dot{z}(0)+\gamma }.$$

## 4. Existence

The proof of theorem 3.1 is based on the Leray–Schauder fixed point theorem [40]. We shall show that the solutions to (3.5) and (3.6) satisfy *a priori* bounds, and we will use them to show compactness of a suitable operator.

**Lemma 4.1.**

*Let* $X_{0}>0$ *and* $X_{1}>0$. *Suppose there exists a solution* z *to (3.5) and (3.6) with data* $X_{0}$ *and* $X_{1}$. *Then, there exists some constant* C *such that* |z(t)|≤C, *for* $t\in [t_{0},t_{2}]$. *Moreover*, C *is only in terms of the parameters and the data*.

**Proof.**

Let $\bar{\beta_{e}}:=\beta_{e}\;\gamma^{-1}$ and let $\tau \in [t_{0},t_{2}]$. Integrating (3.5) over $(t_{0},\tau ),$ we have

$$\dot{z}(\tau )-\dot{z}(t_{0})=\bar{\beta_{e}}(e^{z(t_{0})}-e^{z(\tau )})-\beta_{e}\int_{t_{0}}^{\tau }\;e^{z(s)}\;\text{d}s.$$

Let $t\in [t_{0},t_{2}]$ and let $\delta_{0}(t):=t-t_{0}$. Then, by integrating with respect to $\tau \in (t_{0},t)$, we have

$$z(t)-z(t_{0})-\delta_{0}(t)\;\dot{z}(t_{0})=\bar{\beta_{e}}\left(\delta_{0}(t)\;e^{z(t_{0})}-\int_{t_{0}}^{t}\;e^{z(\tau )}\;\text{d}\tau \right)-\beta_{e}\int_{t_{0}}^{t}\int_{t_{0}}^{\tau }\;e^{z(s)}\;\text{d}s\;\text{d}\tau .$$

Since the exponential function is positive everywhere, we see that

$$0\leq \int_{t_{0}}^{t}\;e^{z(\tau )}\;\text{d}\tau \leq \int_{t_{0}}^{t_{2}}\;e^{z(\tau )}\;\text{d}\tau =X_{0}+X_{1},$$

and consequently

$$0\leq \int_{t_{0}}^{t}\int_{t_{0}}^{\tau }\;e^{z(s)}\;\text{d}s\;\text{d}\tau \leq \delta_{0}(t)(X_{0}+X_{1}).$$

Hence, we see that there exist bounds

4.1 $$z_{0,l}(t)\leq z(t)\leq z_{0,u}(t),$$

for $t\in [t_{0},t_{2}]$, where

4.2 $$z_{0,l}(t):=z(t_{0})+\delta_{0}(t)(\dot{z}(t_{0})+\bar{\beta_{e}}\;e^{z(t_{0})})-\bar{\beta_{e}}(1+\gamma \;\delta_{0}(t))(X_{0}+X_{1})$$

and

4.3 $$\begin{array}{r} z_{0,u}(t):=z(t_{0})+\delta_{0}(t)(\dot{z}(t_{0})+\bar{\beta_{e}}\;e^{z(t_{0})}). \end{array}$$

These bounds depend on z and $\dot{z}$ through their values at $t_{0}$. Since we are interested in uniform bounds for any solution z, we need to show that $z(t_{0})$ and $\dot{z}(t_{0})$ are bounded in terms of the data $X_{0}$ and $X_{1}$. By exponentiation and integration (4.1) over $\left(t_{0},t_{2}\right)$, we have

4.4 $$\int_{t_{0}}^{t_{2}}\;e^{z_{0,l}(t)}\;\text{d}t\leq (X_{0}+X_{1})\leq \int_{t_{0}}^{t_{2}}\;e^{z_{0,u}(t)}\;\text{d}t,$$

and hence, by taking (4.2) into account,

$$\begin{array}{rl} \int_{t_{0}}^{t_{2}}\;e^{z_{0,l}(t)}\;\text{d}t & =e^{z(t_{0})-\bar{\beta_{e}}(X_{0}+X_{1})}\int_{t_{0}}^{t_{2}}\;e^{\delta_{0}(t)(\dot{z}(t_{0})+\bar{\beta_{e}}\;e^{z(t_{0})}-\beta_{e}(X_{0}+X_{1}))}\;\text{d}t\\ & =\frac{e^{z(t_{0})-\bar{\beta_{e}}(X_{0}+X_{1})}}{\dot{z}(t_{0})+\bar{\beta_{e}}\;e^{z(t_{0})}-\beta_{e}(X_{0}+X_{1})}\left(e^{\delta_{0}(t_{2})(\dot{z}(t_{0})+\bar{\beta_{e}}\;e^{z(t_{0})}-\beta_{e}(X_{0}+X_{1}))}-1\right). \end{array}$$

To show that $z(t_{0})$ and $\dot{z}(t_{0})$ are bounded, we proceed by contradiction. Let $\alpha_{0}:=e^{z(t_{0})}$ and $\sigma_{0}:=\dot{z}(t_{0})$, and let

$$f_{0,l}(\alpha_{0},\sigma_{0}):=\frac{\alpha_{0}\;e^{-\bar{\beta_{e}}(X_{0}+X_{1})}}{\sigma_{0}+\bar{\beta_{e}}\alpha_{0}-\beta_{e}(X_{0}+X_{1})}\left(e^{\delta_{0}(t_{2})(\sigma_{0}+\bar{\beta_{e}}\alpha_{0}-\beta_{e}(X_{0}+X_{1}))}-1\right).$$

A standard application of L’Hôpital’s rule yields

$$\lim_{\alpha_{0}\to \infty }\;f_{0,l}(\alpha_{0},\sigma_{0})=\infty \;\text{and}\;\lim_{\sigma_{0}\to \infty }\;f_{0,l}(\alpha_{0},\sigma_{0})=\infty ,$$

where we have fixed $\sigma_{0}$ in the first limit and fixed $\alpha_{0}$ in the second limit, and similarly

$$\lim_{\alpha_{0},\sigma_{0}\to \infty }\;f_{0,l}=\infty .$$

Since (4.4) implies boundedness of $f_{0,l}$, we conclude $\alpha_{0}$ and $\sigma_{0}$ cannot be arbitrarily large and in consequence, $z(t_{0})$ and $\dot{z}(t_{0})$ are bounded from above. Using a similar argument with the right-hand side bound of (4.4) and taking the limits $\alpha_{0}\to 0$ and $\sigma_{0}\to -\infty$, we can conclude that $z(t_{0})$ and $\dot{z}(t_{0})$ are also bounded from below.

Therefore, the bounds (4.2) and (4.3) are independent of the solution z, and depend only on the parameters and the data. ▪

We can now proceed to show that $\dot{z}$ is also uniformly bounded in $\left[t_{0},t_{2}\right]$.

**Lemma 4.2.**

*Let* $X_{0}>0$ *and* $X_{1}>0$. *Suppose there exists a solution* z *to (3.5) and (3.6) with data* $X_{0}$ *and* $X_{1}$. *Then, there exists some constant* $C^{\prime }$ *such that* $|\dot{z}(t)|\leq C^{\prime }$, *for* $t\in [t_{0},t_{2}]$. *Moreover*, $C^{\prime }$ *is only in terms of the parameters and the data*.

**Proof.**

Using the notation introduced in the proof of lemma 4.1, we integrate (3.5) over $\left(t_{0},t\right)$, where $t\in [t_{0},t_{2}]$, to obtain

4.5 $$\dot{z}(t)=\dot{z}(t_{0})-\bar{\beta_{e}}\left(e^{z(t)}-e^{z(t_{0})}\right)-\beta_{e}\int_{t_{0}}^{t}\;e^{z(s)}\;\text{d}s.$$

We can now use the uniform bounds on z, from lemma 4.1, together with the bounds on $z(t_{0})$ and $\dot{z}(t_{0})$ that we obtained in the proof of lemma 4.1, to bound the right-hand side of (4.5). ▪

We can proceed using equations (3.5) and (3.6) and the bounds on z and $\dot{z}$ to obtain uniform bounds on the second derivative, which in turns allows us to obtain uniform bounds for the third derivative by using them in (3.5). Indeed, we can bootstrap this argument to obtain uniform bounds up to the nth derivative, for finite n. We summarize this in the following corollary.

**Corollary 4.3.**

*Let* $n=0,1,\ldots ,\bar{N}$, *with* $\bar{N}<\infty$, *and let* $X_{0}>0$ *and* $X_{1}>0$. *Suppose there exists a solution* z *to* (3.5) *and* (3.6) *with data* $X_{0}$ *and* $X_{1}$. *Then, there exists some constant* $C^{\left(n\right)}$, *depending only on* n, *the data and the parameters, such that* $|z^{\left(n\right)}(t)|<C^{\left(n\right)}$ *for all* $t\in [t_{0},t_{2}]$.

We can now use the uniform bounds on the solutions to study a suitable operator that will allow us to define the solutions of (3.5) and (3.6) as a fixed point. Let

4.6 $$F[z](t):=a_{z}+b_{z}t-\int_{t_{0}}^{t}\int_{t_{0}}^{\tau }\beta_{e}\;e^{z(s)}\left(\frac{\dot{z}(s)}{\gamma }+1\right)\;\text{d}s\;\text{d}\tau ,$$

where $a_{z},b_{z}$ depend on z and are uniquely determined under the constraints

4.7 $$\int_{t_{0}}^{t_{1}}\;e^{F[z](t)}\;\text{d}t=X_{0}\;\mathrm{and}\;\int_{t_{1}}^{t_{2}}\;e^{F[z](t)}\;\text{d}t=X_{1}.$$

We note importantly that a fixed point of F is a solution to (3.5)–(3.6). Therefore, our goal is to apply the Leray–Schauder fixed point theorem to F to show the existence of a solution in $C^{2}(t_{0},t_{2})$. We will first show that F is a compact operator.

**Lemma 4.4.**

*Let* F *be defined as in (4.6). Then* F *is a compact operator in* $C^{2}(t_{0},t_{2})$.

**Proof.**

Let ${\left(z_{n}\right)}_{n\in N_{0}}$ be a sequence of functions bounded in $C^{2}(t_{0},t_{2})$. We see that since

$${\left(F[z_{n}]\right)}^{\left(3\right)}=-\beta_{e}{\dot{z}}_{n}\;e^{z_{n}}\left(\frac{{\dot{z}}_{n}}{\gamma }+1\right)-\beta_{e}\;e^{z_{n}}\frac{{\ddot{z}}_{n}}{\gamma },$$

we have ${\left(F[z_{n}]\right)}^{\left(3\right)}$ is also uniformly bounded, since ${\left(z_{n}\right)}_{n}$ is uniformly bounded in $C^{2}(t_{0},t_{2})$. Therefore, the Arzela–Ascoli theorem implies that F is a compact operator in $C^{2}(t_{0},t_{2})$. We note that to apply the Arzela–Ascoli theorem in $C^{2}(t_{0},t_{2})$ suffices to show uniform boundedness of the third derivative, since then we also have equicontinuity of the second derivative; this is analogous to using uniform boundedness of the first derivative to show equicontinuity of the sequence of functions. ▪

With this set-up, in particular that F is compact in $C^{2}(t_{0},t_{2})$, we can now finish the proof of theorem 3.1.

**Proof of theorem 3.1.**

In order to apply the Leray–Schauder fixed point theory, we now need to show that the set

4.8 $$\left\{z\in C^{2}(t_{0},t_{2}):z=\kappa F[z]\right\}$$

is bounded for all κ∈[0,1]. It is easy to see, by linearity of the derivative and the integral, that a fixed point of κF corresponds to a fixed point of F with a change of parameters, namely, by replacing $\beta_{e}$ by $\kappa \beta_{e}$. Therefore, any function z in (4.8) is a solution to (3.5) and (3.6) with a suitable parameter, and as shown in lemma 4.1, is bounded in $C^{2}(t_{0},t_{2})$. Thus the Leray–Schauder fixed point theorem implies the existence of a fixed point for F, which, as already mentioned, corresponds to a solution of (3.5)–(3.6). ▪

## 5. Uniqueness

Now that we have proven that there exists a solution to (3.5)–(3.6), we now want to demonstrate the uniqueness of a solution to (3.5)–(3.6). We employ a standard contradiction argument for boundary value problems by showing that two different solutions, which satisfy the same boundary conditions, must be the same.

**Proof of theorem 3.2.**

Let x(t) and y(t) be two solutions to (3.5) and (3.6) satisfying the same data conditions, that is, for m=0,1 we have

$$X_{m}=\int_{t_{m}}^{t_{m+1}}\;e^{x(s)}\;\text{d}s=\int_{t_{m}}^{t_{m+1}}\;e^{y(s)}\;\text{d}s.$$

Consequently, this means that

5.1 $$\int_{t_{m}}^{t_{m+1}}\;e^{x(s)}-e^{y(s)}\;\text{d}s=0.$$

Let D(t):=x(t)−y(t) and $E(t):=e^{x(t)}-e^{y(t)}$. By taking the difference of (3.5) for each solution, we have

5.2 $$\ddot{D}=-\beta_{e}\left(E+\frac{\dot{E}}{\gamma }\right).$$

To simplify the presentation, we set $\bar{\beta_{e}}:=\beta_{e}\;\gamma^{-1}$ and denote

$$F(t):=\dot{D}(t)+\bar{\beta_{e}}E(t)-\dot{D}(t_{0})-\bar{\beta_{e}}E(t_{0}).$$

Using this notation, (5.2) is equivalent to

5.3 $$\dot{F}+\beta_{e}E=0.$$

Let $t\in [t_{0},t_{2}]$. Integrating (5.3) with respect to $s\in (t_{0},t)$ gives

5.4 $$F(t)+\beta_{e}\int_{t_{0}}^{t}E(s)\;\text{d}s=0.$$

Moreover, by (5.1) and (5.4), one can see that

5.5 $$F(t_{1})=F(t_{2})=0.$$

We now proceed by contradiction to show that $D(t_{0})=0$. Without loss of generality, assume that $D(t_{0})>0$. This gives us that $E(t_{0})>0$ by definition and $\dot{F}(t_{0})<0$ by (5.3). Considering this, (5.5) and the fact that x, $y\in C^{2}(t_{0},t_{2})$, we have that there exists $\tau \in (t_{0},t_{1}]$ such that F(τ)=0 and F(t)<0 for all $t\in (t_{0},\tau )$. In particular, this means that, by integrating F with respect to $s\in (t_{0},\tau )$, we have

5.6 $$\int_{t_{0}}^{\tau }F(s)\;\text{d}s<0$$

and, setting t=τ in (5.4), we have

5.7 $$\int_{t_{0}}^{\tau }E(s)\;\text{d}s=0.$$

Then, by integrating (5.4) with respect to $t\in (t_{0},\tau )$ and using (5.6), we obtain

$$0=\int_{t_{0}}^{\tau }F(t)\;\text{d}t+\beta_{e}\int_{t_{0}}^{\tau }\int_{t_{0}}^{t}E(s)\;\text{d}s\;\text{d}t<\beta_{e}\int_{t_{0}}^{\tau }\int_{t_{0}}^{t}E(s)\;\text{d}s\;\text{d}t=0,$$

where the last equality is a consequence of Fubini’s theorem and (5.7). Therefore, we have a contradiction. One notices that, using the same approach, we can also conclude that $D(t_{1})=0$ due to (5.5). By integrating (5.4) with respect to $t\in (t_{0},t_{1})$, we have that

$$D(t_{1})+\bar{\beta_{e}}\int_{t_{0}}^{t_{1}}E(t)\;\text{d}t+\beta_{e}\int_{t_{0}}^{t_{1}}\int_{t_{0}}^{t}E(s)\;\text{d}s\;\text{d}t=D(t_{0})+(t_{1}-t_{0})(\dot{D}(t_{0})+\bar{\beta_{e}}E(t_{0})),$$

which, by noting that $D(t_{0})=0$ implies that $E(t_{0})=0$ and using (5.1), gives us

$$\dot{D}(t_{0})=0.$$

Standard ODE theory implies that, since $x(t_{0})=y(t_{0})$, $\dot{x}(t_{0})=\dot{y}(t_{0})$ and x, $y\in C^{2}(t_{0},t_{2})$, we have x(t)=y(t) for all $t\in [t_{0},t_{2}]$. ▪

## 6. Numerical examples

Now that we have demonstrated existence and uniqueness, we can build a numerical algorithm to approximate the solution to (3.5)–(3.6). We set up the numerical problem as follows: given $X_{0}$, $X_{1}$, $\beta_{e}$ and γ, we look to find the function z that satisfies (3.5)–(3.6). We use the shooting method to numerically approximate the solution to (3.5), see for example [41] for details. We use a superscript 0 to denote the initial approximation to z and a superscript ε to denote the converged solution from the shooting method.

**Figure 2.**
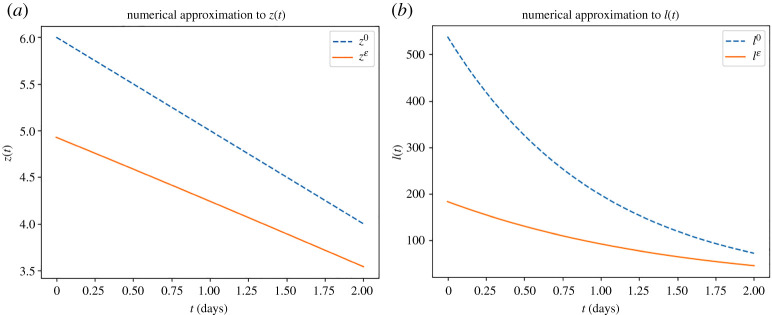
Demonstration of the numerical approach to approximate the solutions to (3.5) and (3.6) in the case of the number of detected cases is reducing. (*a*) Comparison of the initial guess for z and the converged numerical approximation for $z^{\varepsilon }$. (*b*) Comparison of the transformed initial guess for I and the transformed converged numerical approximation for $I^{\varepsilon }$. (Online version in colour.)

For our first example, we look at the case where the number of detected cases is reducing. We set $\beta_{e}=8\times 10^{-4}$, γ=1 and use the data $X_{0}=100$ and $X_{1}=50$ with $t_{0}=0$, $t_{1}=1$ and $t_{2}=2$. For the shooting method, we use an initial guess of $z^{0}(0)=6$, ${\dot{z}}^{0}(0)=-1$, depicted by the dashed blue line in figure 2*a*, which results in

$$\int_{0}^{1}\;e^{z^{0}(s)}\;\text{d}s\approx 255.00079468571883\;\text{and}\;\int_{1}^{2}\;e^{z^{0}(s)}\;\text{d}s\approx 93.80954898172891.$$

The resulting initial conditions from the shooting method are $z^{\varepsilon }(0)=4.92579565$ and ${\dot{z}}^{\varepsilon }(0)=-0.66986594$, with the resulting function $z^{\varepsilon }$ depicted by the continuous orange line in figure 2*a*. Even although we can typically gain knowledge of the total population N, through for example a population census, as previously mentioned we cannot extract β from $\beta_{e}$ due to the under-reporting parameter r. However, for the purpose of demonstration, we fix r=0.75 and N=1000 to allow us to demonstrate $I^{0}$ and $I^{\varepsilon }$, the number of infectious people transformed from $z^{0}$ and $z^{\varepsilon }$, respectively. Figure 2*b* depicts the transformed initial guess $I^{0}$ as the dashed blue line and depicts the transformed converged solution $I^{\varepsilon }$ as the continuous orange line.

**Figure 3.**
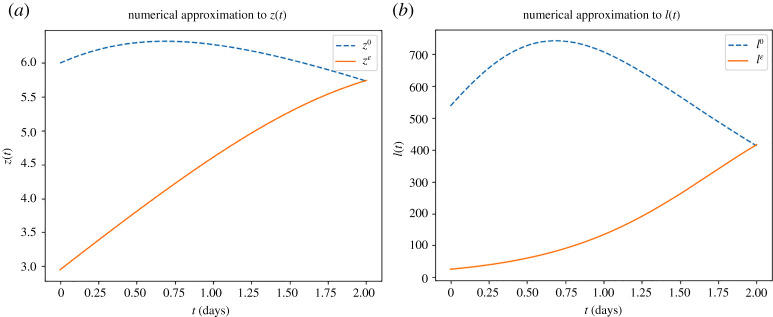
Demonstration of the numerical approach to approximate the solutions to (3.5) and (3.6) in the case of the number of detected cases is increasing. (*a*) Comparison of the initial guess for z and the converged numerical approximation for $z^{\varepsilon }$. (*b*) Comparison of the transformed initial guess for I and the transformed converged numerical approximation for $I^{\varepsilon }$. (Online version in colour.)

For our second example, we look at the case where the number of detected cases is increasing. We set $\beta_{e}=2\times 10^{-3}$, γ=1 and use the data $X_{0}=50$ and $X_{1}=200$ with $t_{0}=0$, $t_{1}=1$ and $t_{2}=2$. For the shooting method, we use an initial guess of $z^{0}(0)=6$ and ${\dot{z}}^{0}(0)=1$, depicted by the dashed blue line in figure 3*a*, which results in

$$\int_{0}^{1}\;e^{z^{0}(s)}\;\text{d}s\approx 517.9831215391448\;\text{and}\;\int_{1}^{2}\;e^{z^{0}(s)}\;\text{d}s\approx 423.89217327827055.$$

The resulting initial conditions from the shooting method are $z^{\varepsilon }(0)=2.9481878$ and ${\dot{z}}^{\varepsilon }(0)=1.75958188$, with the resulting function $z^{\varepsilon }$ depicted as the continuous orange line in figure 3*a*. Again, we fix r=0.75 and N=1000 to allow us to demonstrate $I^{0}$ and $I^{\varepsilon }$ in figure 3*b*, whereby the transformed initial guess $I^{0}$ is depicted by the dashed blue line and the transformed converged solution $I^{\varepsilon }$ is depicted by the continuous orange line.

## 7. Conclusion

In this publication, we have demonstrated how to derive the observational model given data that describes the infectious compartment I, shown that a solution to this formulation exists and that the solution is unique. We have then gone on to demonstrate an algorithm to approximate the solution to the nonlinear observational model. While it is standard these days for more complex and descriptive compartmental models to be analysed and used in practice, this study provides a first illustration of the analysis for the novel boundary value approach, which we intend to build upon to combat the issues outlined in §§2 and 3. It is not hard to reason that using two data points is not enough to provide accurate forecasting capabilities for the short and medium term, but this result combined with parameter estimation with more data points and a model selection approach will shed light on how one can use this approach.

Now that we have guaranteed that the solution to the observational model is unique, given some parameters, we next look to answer the questions of the identifiability of the parameters from the data and how to appropriately deal with noise in the data. That is to say, how much data do we need to be able to uniquely identify the parameters $\beta_{e}$ and γ as well as z? These are questions of particular importance as understanding what we can identify, and the accuracy of the estimations, helps us to understand the scenario-based forecasting capabilities of the mathematical model. Understanding this and therefore the underlying assumptions needed to produce forecasting results, given the limelight of compartmental models in recent history, will allow for a confident and trustworthy exchange of knowledge between mathematical modellers and those with research questions on infectious diseases, such as local government or healthcare providers.

## Data Availability

This article has no additional data.
